# The Vicissicaudata revisited – insights from a new aglaspidid arthropod with caudal appendages from the Furongian of China

**DOI:** 10.1038/s41598-017-11610-5

**Published:** 2017-09-11

**Authors:** Rudy Lerosey-Aubril, Xuejian Zhu, Javier Ortega-Hernández

**Affiliations:** 1 Palaeoscience Research Centre, School of Environmental and Rural Science, University of New England, Armidale, NSW 2351 Australia; 2 State Key Laboratory of Palaeobiology and Stratigraphy, Nanjing Institute of Geology and Palaeontology, Chinese Academy of Sciences, Nanjing, 210008 China; 3 Department of Zoology, University of Cambridge, Cambridge, CB2 3EJ UK

## Abstract

Cambrian marine ecosystems were dominated by arthropods, and more specifically artiopods. Aglaspidids represent an atypical group amongst them, not the least because they evolved and rapidly diversified during the late Cambrian, a time interval between the two diversification events of the Early Palaeozoic. Recent phylogenetic analyses have retrieved aglaspidids within the Vicissicaudata, a potentially important, but difficult to define clade of artiopods. Here we describe a new aglaspidid from the Furongian Guole Konservat-Lagerstätte of South China. This taxon displays a pretelsonic segment bearing non-walking appendages, features as-yet known in all vicissicaudatans, but aglaspidids. A new comprehensive phylogenetic analysis provides strong support for the legitimacy of a monophyletic clade Vicissicaudata, and demonstrates the pertinence of new characters to define Aglaspidida. It also motivates important changes to the systematics of the phylum, including the elevation of Artiopoda to the rank of subphylum, and the establishment of a new superclass Vicissicaudata and a new aglaspidid family Tremaglaspididae. Two diversification pulses can be recognized in the early history of artiopods – one in the early Cambrian (trilobitomorphs) and the other in the late Cambrian (vicissicaudatans). The discrepancy between this pattern and that traditionally depicted for marine invertebrates in the Early Palaeozoic is discussed.

## Introduction

**Figure 1:**
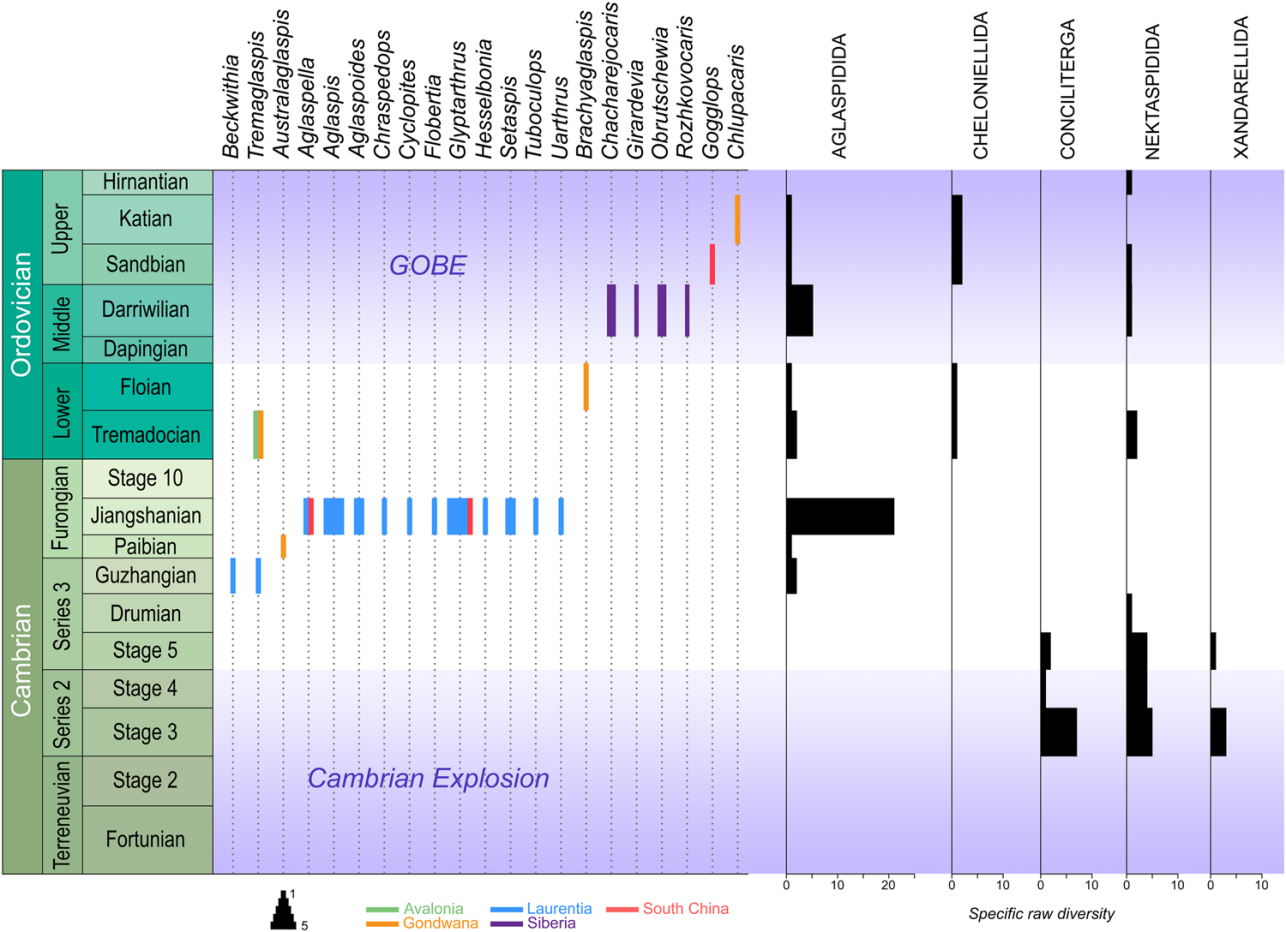
Stratigraphic distribution, palaeobiogeography, and diversity of aglaspidids and other artiopods in the Lower Palaeozoic. Most aglaspidid genera (on the left) are known from Jiangshanian strata of the Upper Mississippi Valley (Laurentia). *Quasimodaspis brentsae* Waggoner^56^ is not represented on this figure due to uncertainties about its age. Aglaspidids are atypical amongst artiopods (on the right), for they first evolved and rapidly diversified during the time interval in-between the Cambrian Explosion and the Great Ordovician Biodiversification Event (GOBE).

The Aglaspidida is a group of early Palaeozoic marine arthropods that has attracted considerable attention by virtue of possessing a number of atypical features compared to other contemporaneous members of the phylum Arthropoda. For instance, aglaspidids are amongst the rare arthropods, along with bradoriids, phosphatocopids and a few other taxa^1^, to have evolved a (lightly) phosphatised exoskeleton^2^. Partly due to this rare anatomical trait and despite a shorter stratigraphical range, their documented diversity (at least 21 genera) is also greater than that of any arthropod clade of comparable – or higher – taxonomic rank that inhabited Cambro-Ordovician seas (e.g. Conciliterga, Marrellomorpha, Nektaspidida, Petalopleura), with trilobites as the only notable exception (c. 4020 genera, including c. 3170 Cambro-Ordovician ones; Adrain, pers. com. 2017). More interesting is the absence of legitimate aglaspidids in Cambrian Stage 3 to Drumian marine deposits, despite an over-representation of Konservat-Lagerstätten in these strata^3, 4^. Indeed, their fossil record suggests that they first appeared in Laurentia during the Guzhangian^5^ (late Cambrian Epoch 3) and rapidly diversified to reach a peak in the Jiangshanian (Furongian; Fig. 1). By then, their palaeogeographical distribution had become global, as evidenced by occurrences in Gondwana (Tasmania^6^), Laurentia (Canada^2^, USA^7, 8^), South China^9^, and possibly Siberia^10^ (the age of the last material is poorly constrained, hence its absence in Fig. 1). In the Ordovician, the group is known with confidence from the Tremadocian of Avalonia^11, 12^ (Wales), the Tremadocian^13^ and Floian^14^ of Gondwana (Morocco), the Darriwilian of Siberia (ref. 15 and references therein), and the Sandbian of South China^16^; the youngest representative as-yet described is *Chlupacaris* ^17^ from the Katian of Morocco. In summary, aglaspidids possess a particularly atypical palaeodiversity pattern – their first appearance is after the Cambrian Explosion and their main (first?) radiation was during the Furongian, that is to say well before the onset of the Great Ordovician Biodiversification ‘Event’ (GOBE; Fig. 1).

The evolutionary origin of the group remains problematic. *Kwanyinaspis maotianshanensis* Zhang and Shu^18^, from the Cambrian (Stage 3) Chengjiang Lagerstätte, was first tentatively assigned to the Aglaspidida, but this view has not gained wide acceptance^17^. Indeed, *Kwanyinaspis* has been recovered in various positions within Artiopoda in subsequent cladistic analyses, but never within Aglaspidida^19–24^. Recent phylogenetic analyses have resolved aglaspidids as part of a larger clade known as Vicissicaudata^22^, which also includes the Burgess Shale (Cambrian Age 5) arthropods *Emeraldella* and *Sidneyia*, and the Early Ordovician–Early Devonian cheloniellids^20, 22, 25^. However, this group has been essentially recovered when using character weighting and identifying precise synapomorphic characters for it has proved challenging^2^.

The aim of this contribution is to provide a substantial update on the current understanding of the origin and evolution of Aglaspidida within the phylogenetic context of other major groups of Lower Palaeozoic artiopods. In addition to revising the definition and applicability of Vicissicaudata, we also describe a new aglaspidid arthropod from the Furongian Guole Konservat-Lagerstätte of South China, which provides new insights into the evolution of tagmosis in the Aglaspidida. We then combine our new data with that of recent systematic descriptions of closely related taxa into a comprehensive phylogenetic analysis, in order to examine their implications for the relationships between major artiopodan clades. Our findings support the legitimacy of Vicissicaudata as a monophyletic clade, leading us to propose a more precise definition for this group, and also allow to formalize the internal systematic classification within Aglaspidida.

## Vicissicaudata – an elusive concept

Cambrian marine faunas were dominated by members of Artiopoda (*sensu* ^26^), a large clade comprising the highly diverse trilobites as its most familiar representatives, but also including several arthropod groups with a more restricted fossil records (e.g. Aglaspidida, Cheloniellida, Conciliterga, Nektaspidida, Xandarellida). Chelicerates were also retrieved as part of this clade in some cladistic analyses^27–30^ (all these studies used matrices largely derived from ref. 27), while in others they constitute a distinct clade with no close relationships with artiopods^22–24^. If the last hypothesis is correct, Artiopoda would represent a diverse, yet completely extinct branch of the arthropod tree. The case of chelicerates put aside, two main artiopodan groups have been recovered in several recent cladistic analyses: the Trilobitomorpha and the Vicissicaudata. The former clade regroups concilitergans, nektaspidids, trilobites, xandarellids and various other taxa, and has gained wide acceptance over the last fifteen years^19, 21–24, 27, 28, 31, 32^ (‘clade NN1’ of ref. 23; ‘clade G’ of ref. 32). Vicissicaudata – a clade essentially composed of aglaspidids, cheloniellids, and the Burgess Shale arthropods *Emeraldella* and *Sidneyia* – has proved more elusive and difficult to define.

Lerosey-Aubril *et al*.^2^ reviewed the concept of Vicissicaudata and its limitations, with the main conclusions summarized as follows: 1) close phylogenetic relationships between aglaspidids, cheloniellids, *Emeraldella* and *Sidneyia* ^17, 20, 22, 24, 25, 28^, or at least some of these taxa^23, 32, 33^, have been repeatedly hypothesized in the past; 2) these taxa constitute a distinct clade in several phylogenetic analyses, essentially recovered when using implied weight parsimony^20, 22, 24, 25, 28^; 3) this clade, referred to as ‘clade 5’ in early studies and formally named Vicissicaudata in ref. 22, was originally recovered as allied to chelicerates and megacheirans^20, 25^, but more recent analyses have retrieved it within the Artiopoda, either as the sister group of Trilobitomorpha^22, 24^, or as the sister group of Chelicerata in studies regarding chelicerates as artiopods^27, 28^; 4) there is no consensus regarding the relationships within the Vicissicaudata^22^; 5) identifying characters defining this clade has proved challenging, with Cotton and Braddy^25^ initially proposing three of them, Edgecombe *et al*.^20^ two, and finally Ortega-Hernández *et al*.^22^ only a single one – the presence of a ‘postabdomen expressed as posterior segments lacking walking legs’ (see ref. 2 for comments on this character).

A recently described aglaspidid species from Furongian (Jiangshanian) strata of the McKay Group (Canada) has proved particularly informative with regards to the origin of aglaspidids and their relationships with other vicissicaudatans^2^. This taxon, *Glypharthrus magnoculus*, exhibits all the characteristics of a Cambrian-type aglaspidid (*sensu* ^22^; equivalent to family Aglaspididae, as redefined below): a flat body with wide (tr.) pleural regions, a cephalon bearing acute genal angles, cephalic furrows, and prominent eyes, and a trunk terminated by a long terminal spine. However, it differs from all Cambrian aglaspidids by the presence of twelve, rather than eleven trunk tergites. This extra trunk tergite differs from more anterior ones in being particularly long (sag.) and virtually devoid of pleurae. A similarly differentiated posteriormost trunk tergite – the pretelsonic segment – is also known in cheloniellids, *Emeraldella*, and *Sidneyia* (the last taxon actually possesses a posterior trunk region made of two such segments). This led Lerosey-Aubril *et al*.^2^ to propose the presence of this morphologically differentiated, terminal trunk tergite as a more precise character for the diagnosis of Vicissicaudata. Interestingly, the spine projecting from the trunk posteriorly in *G. magnoculus* is also different from the tailspine of most Cambrian-type aglaspidids, as it does not possess an anterior expansion (‘large base’). As argued by Lerosey-Aubril *et al*.^2^, the co-occurrence of a twelfth pretelsonic-like trunk tergite and a simple terminal spine in this species strongly supports the hypothesis of a composite nature for the aglaspidid tailspine. This hypothesis, first proposed by Van Roy^17^, states that the tailspine of aglaspidids is the result of the fusion of a spiniform telson and (at least) one pretelsonic segment, similar to that of other vicissicaudatans, during the early evolution of the group. The discovery of a new aglaspidid species in the Furongian of Guangxi, which displays a pretelsonic segment with caudal appendages, provides decisive support for this hypothesis.

## Results

### Geological Setting

**Figure 2:**
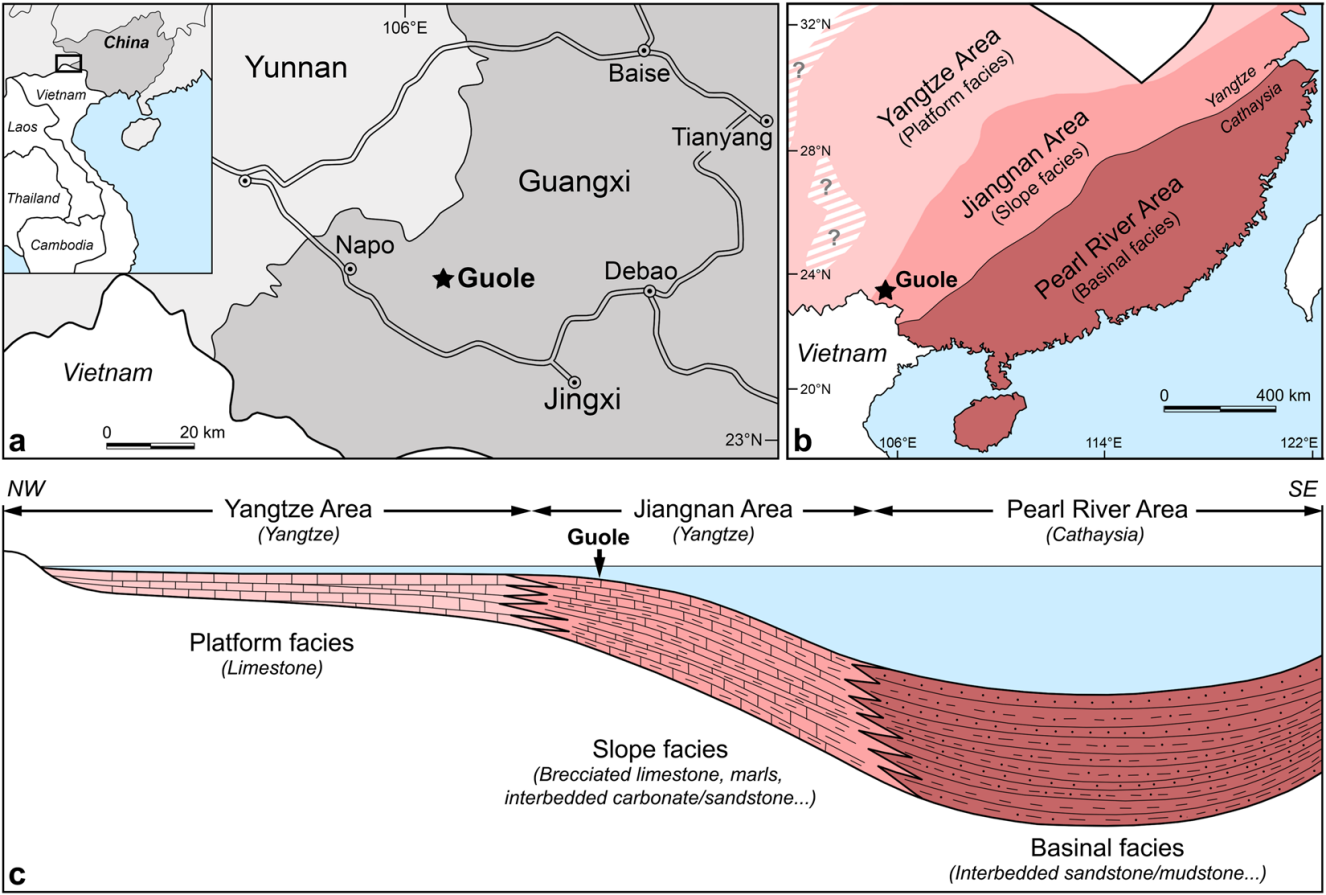
Location and depositional environment of the Jiangshanian strata of the Sandu Formation exposed in the Guole area, Guangxi, South China. (**a**) location of Guole area, c. 40 km N of the Vietnamese border. Map created using Adobe Photoshop CS6 (http://www.adobe.com/products/photoshop.html). (**b**) late Cambrian lithofacies belts in SE China (modified after ref. 39); note the location of Guole within the Jiangnan area (slope lithofacies belt). (**c**) depositional environments associated with the upper Cambrian lithofacies belts of SE China; note that the Jiangshanian strata in the Guole area were probably deposited in the uppermost part of the continental slope.

The fossils described herein were collected from marls (calcareous mudstone) of the Sandu Formation in the vicinity of Guole Township, Jingxi County, western Guangxi Zhuang Autonomous Region, South China (Fig. 2a). Aglaspidid remains were found at two localities. One is a small quarry located c. 2 km N of Guole Township, where specimens NIGPAS 165043 and 165044 were recovered, along with the only known specimen (NIGPAS 157028) of another aglaspidid species, *Aglaspella sanduensis* Lerosey-Aubril *et al*.^9^. A fragment of a cephalon, possibly belonging to a third representative of this group (Fig. 3c in ref. 34), was also found in close proximity to this small quarry. The second excavation site is a roadcut located c. 1 km NW of Guole Township (c. 1 km SW of the quarry); it exposes a c. 3 m-thick section that has yielded the holotype specimen (NIGPAS 165042).

**Figure 3:**
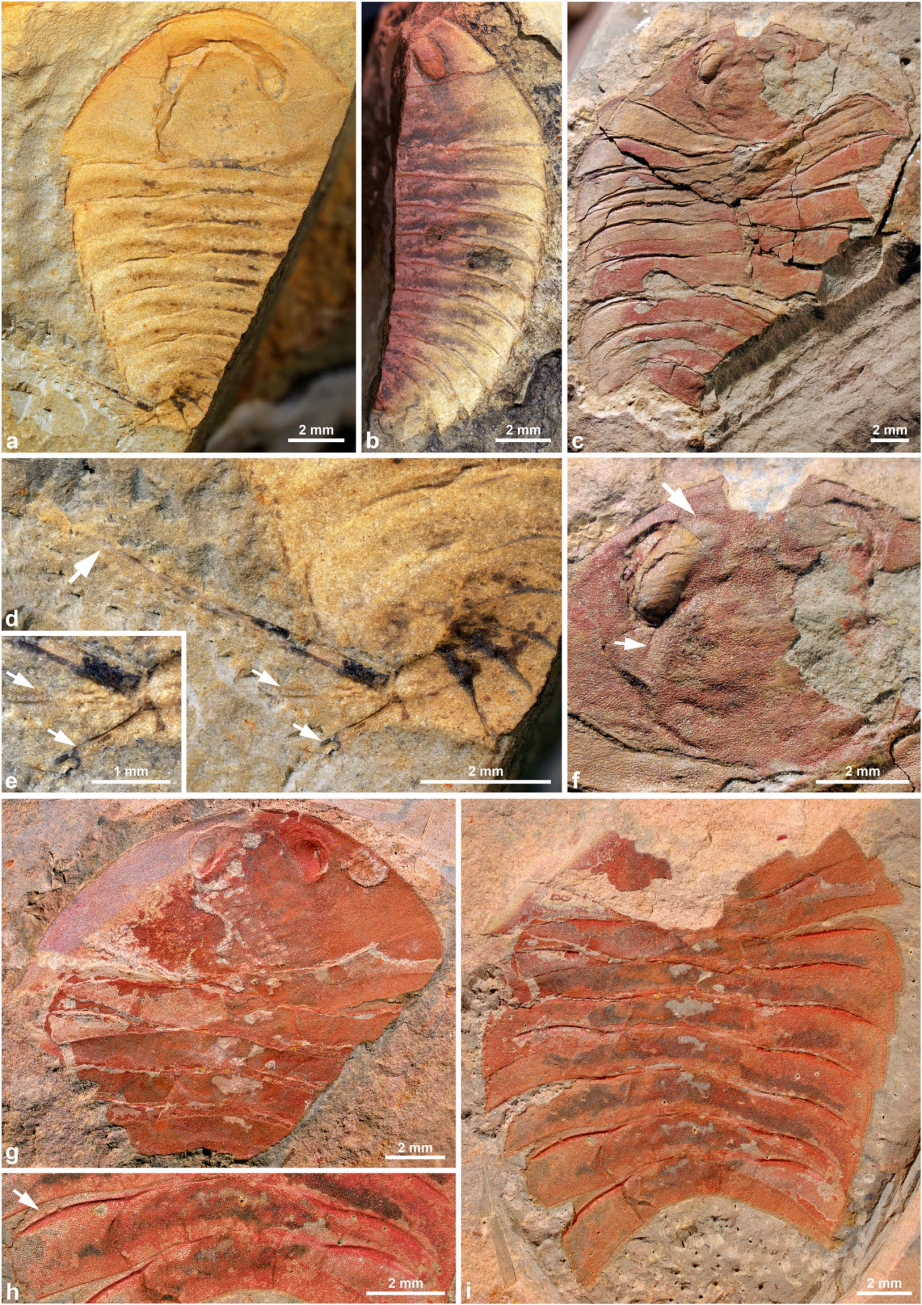
*Glypharthrus trispinicaudatus* sp. nov. from the Jiangshanian (Furongian) Sandu Formation, Guole area, Guangxi, South China. All specimens are in dorsal view with anterior facing to the top. (**a**,**b**,**d,e**) NIGPAS 165042 (holotype), part and counterpart of an almost complete, articulated dorsal exoskeleton, photographed immersed in dilute ethanol. (**a**,**d**,**e**) counterpart (mostly an external mould). (**a**) general view. (**d,e**) detail of posterior trunk region, showing pretelsonic segment bearing a pair of furcal rami (small arrows) and a spiniform telson (large arrow). (**b**) part (dorsal exoskeleton; mirrored). (**c**,**f**) NIGPAS 165043, almost complete, largely articulated dorsal exoskeleton, photographed dry. (**c**) general view. (**f**) detail of cephalon showing the eye abutting the glabellar region (small arrow) and merging with the rest of the exoskeleton anteriomedially (large arrow), and the pitted surface. (**g**,**h**,**i**) NIGPAS 165044, part and counterpart of an almost complete, largely articulated dorsal exoskeleton, photographed dry (**g,i**) or immersed under dilute ethanol (**h**). (**g**) counterpart (mirrored). (**h**) part, detail of T7, showing the articulating ridge separating the articulating platform (arrow) from the posterior part of the tergite. (**i**) part, general view.

The Sandu Formation was formally described in the eponymous section in Sandu County (Guizhou Province), c. 330 km NE of Jingxi County, but the Furongian deposits of Guole area are traditionally assigned to this lithostratigraphic unit due to striking lithological and palaeontological (e.g. trilobites) similarities^34, 35^ (*contra* ^36^). These similarities are explained by the fact that the two localities are located within the Jiangnan Area, a transitional facies belt dominated by calcareous mudstone, which separated the shallow-water, carbonate platform of the Yangtze Area (Yangtzean part of South China) from the deep-water, fine-grained siliciclastic deposits of the Pearl River Area (i.e. the Nanhua Basin; Cathaysian part of South China) during most of the Cambrian period (Fig. 2b,c). Han *et al*.^36^ hypothesized that the Furongian strata outcropping in the Guole area (their ‘Guole Formation’) have been deposited in shallow-water shelf setting, while Feng *et al*.^37^ interpreted the lithofacies as indicative of basinal environmental conditions. However, if the interpretation of the Jiangnan Area as a transitional facies belt separating the platform (NW) and basinal (SE) facies is correct, a depositional environment in the uppermost part of the continental slope appears more likely for these strata (Fig. 2c)^38, 39^. The Furongian succession near Guole is thought to reach 2000 metres in thickness, but its stratigraphy is in fact poorly known due to limited exposures – for instance, its contact with older rocks is rarely observed. Its upper part is uncomformably overlain by Lower Devonian dolomites (Huangqiongshan Formation).

The Furongian strata outcropping in the Guole area have yielded a diverse marine macroscopic fauna recently reviewed by Zhu *et al*.^34^, who introduced the name ‘Guole Biota’ for it. The ‘shelly’ components include trilobites^36, 40–43^ (at least 21 genera), echinoderms^44–47^ (8 genera), brachiopods^48^ (6 genera), agnostid arthropods^34^ (4 genera), and at least three hyolithid taxa^34, 36^. Interestingly, remains of weakly- or non-biomineralizing organisms have also been recovered from these deposits^34^: a specimen of the putative cnidarian *Sphenothallus*, a pair of arthropod appendages of unknown affinities, the trunk of a new species of mollisoniid-like arthropod, five specimens of aglaspidids (including the three described herein) belonging to two, possibly three different taxa, c. 20 valves of a *Carnarvonia*-like ‘bivalved arthropod’, c. 15 rather complete specimens of a palaeoscolecid worm, and various fragments of graptolites (three species) and algae. According to the trilobite fauna, this biota is Jiangshanian in age (equivalent to the *Probinacunaspis nasalis-Peichiashania hunanensis* Zone of northwestern Hunan^43^).

### Preservation

**Figure 4:**
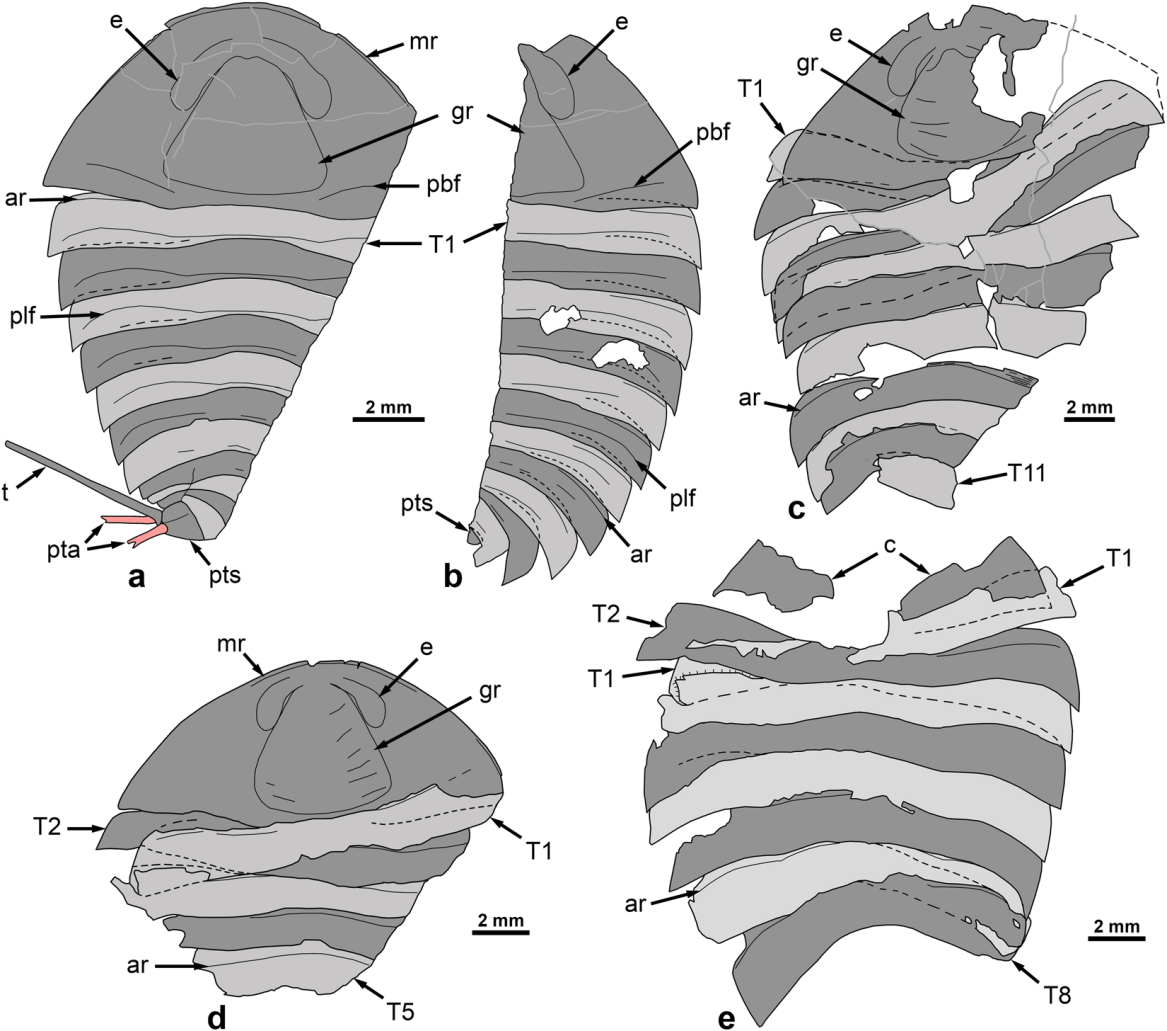
*Glypharthrus trispinicaudatus* sp. nov. from the Jiangshanian (Furongian) Sandu Formation, Guole area, Guangxi, South China. (**a**–**e**) interpretative drawings. (**a**,**b**) part and counterpart of NIGPAS 165042 (holotype). (**c**) NIGPAS 165043. (**d**,**e**) part and counterpart of NIGPAS 165044. Odd and even numbered trunk tergites are in light grey and dark grey, respectively, to facilitate their identification. Abbreviations: ar, articulating ridge; c, cephalon; e, eye; gr, glabellar region; mr, marginal rim; pbf, posterior border furrow; plf, trunk pleural furrow; pta, pre-telsonic appendages (furcal rami); pts, pre-telsonic segment; T(1, 2, 5, 8 or 11), trunk tergite (1, 2, 5, 8 or 11); t, telson.

The specimens represent almost complete dorsal exoskeletons, which were carefully prepared with a needle (Figs 3 and 4). The holotype (NIGPAS 165042, part and counterpart; Figs 3a,b,d,e and 4a,b) is articulated and bears a pair of caudal appendages *in situ*, which suggests that it represents a carcass. In the other two specimens, the posteriormost region of the body is missing (Fig. 3c,i and 4c,e). However, trunk tergites are mostly articulated, except anteriorly where they show a degree of displacement relative to each other and to the cephalon, attesting to the decay of the arthrodial membranes initially connecting them; based on this pattern of disarticulation, we interpret these specimens as freshly moulted exoskeletons. The preferential displacement of the anteriormost trunk tergites and cephalon could be explained by lateral movements of the head, while the animal wriggled out of its old exoskeleton. However, nothing is known of the moulting behaviour of aglaspidids and therefore, determining whether this feature is truly indicative of the presence of a moulted exoskeleton, rather than a carcass cannot be ascertained yet.

All three specimens show clear evidence of dorsoventral flattening, essentially in the forms of compression wrinkles. Some areas of the exoskeleton, such as the eyes and to a lesser extent, the glabellar region still exhibit some relief. The cephalon of the holotype also shows a few cracks apparently resulting from flattening (Figs 3a and 4a), but that of the other two specimens do not, suggesting that they had a purely ductile behaviour in agreement with them being freshly moulted exoskeletons. In any case, the essentially ductile, rather than brittle behaviour in response to compression of the cuticle of this aglaspidid strongly suggests that it was at best weakly biomineralized, if at all^2^. EDS analyses revealed that the material preserving the dorsal exoskeleton is composed of O, Si and Al, along with minor amounts of Fe and Mg – no Ca or P ions were detected. This composition is suggestive of a clay mineral, which has likely replaced the original cuticle during diagenesis. A film of dark dust-like material covered the holotype specimen when the rock containing it was cracked opened, and rapidly dispersed thereafter – this might have represented an intensively weathered superficial (biomineralized?) layer of cuticle.

### Description

The following abbreviations are used hereafter: exs., exsagittally, sag., sagittally, T, trunk tergite, tr., transverse. The three specimens recovered in the Guole area represent a new species of *Glypharthrus*, *G. trispinicaudatus* sp. nov. The holotype, and most complete specimen, is 21 mm in total length (sag.; c. 16 mm, telson excluded) and 5.5 mm in maximum half-width (tr.; Figs 3a,b,d,e and 4a,b). The other two individuals are c. 30% (NIGPAS 165044; Figs 3g–i and 4d,e) and 40% (NIGPAS 165043; Figs 3c,f and 4c) larger than the holotype, as estimated from their cephalic half-width (tr.). In the holotype (Figs 3a and 4a), the cephalon represents c. 37% of body length (telson excluded) and is semi-circular in outline [length (sag.)/maximum width (tr.) ratio: 0.49]. It appears broadly semi-elliptical in outline in the other two specimens (Figs 3c,g and 4c,d), but this might be related to greater compaction. The posterior margin gently curves forwards abaxially, where it intersects with the lateral margins to form acute genal angles. The cephalon is bordered anterolaterally by a narrow marginal rim, which progressively merges with the lateral margins posterolaterally before reaching the genal angles (Figs 3a and 4a). Wide, shallow, sigmoidal posterior furrows run from the posterior margin of the glabellar region (visible in holotype only) towards the lateral cephalic margins without reaching them, thus delimiting posterior borders that widen (exs.) abaxially (Figs 3a,b and 4a,b). Anteriorly, the marginal rim is separated from the dorsal eyes by a particularly narrow preocular field, which represents c. 17% of cephalic total length (sag.) in the holotype, and apparently even less than that in the other two individuals. The sessile dorsal eyes are narrow (tr.), elongate (exs.) and kidney-shaped. They are rather high posteriorly, but extend anteromedially as robust ‘eye ridges’, which progressively lower until merging with the rest of the cephalon some distance from the sagittal axis. The outline of a glabellar region is obvious in all three specimens, although marked by slight breaks in slope of the exoskeleton and wrinkles, rather than actual furrows (Fig. 3f). This glabellar region has a subtriangular outline, occupies c. three-fourths of the cephalic length (sag.) and a third of the maximum cephalic width (tr.), and exhibits slightly angular (Figs 3a and 4a) to rounded (Figs 3c,g and 4c,d) posterolateral corners and a convex backwards posterior margin that almost reaches the posterior margin of the cephalon medially.

The trunk region comprises twelve tergites (T1–12) that are perfectly articulated in the holotype, despite the flexure to the left of the posteriormost part (Figs 3a,b and 4a,b), while they show some degree of displacement in the other two specimens (Figs 3c,i, and 4c,e; see above). It is semi-elliptical in outline and gently, but increasingly narrowing (tr.) posteriorly from c. 95 percent of maximum cephalic width (tr.) at T1 to less than 15 percent of it at T12. T1 is subrectangular in outline, with a length (exs.) representing c. 15% of the width (tr.). It bears a thin ridge that runs subparallel to its entire anterior margin, and separates an articulating platform anteriorly (c. 15–20% of tergite length, exs.) from the rest of the tergite (see Fig. 3h for similar structures in T7). The anterior margin sharply curves backwards abaxially to become the lateral margins, which form acute posterolateral angles together with the essentially straight and transverse posterior margin. A faint anteromedial deflection of the posterior margin indicates the differentiation of an originally raised axial region (see also Fig. 3h,i). A wide and shallow transverse furrow extends throughout the tergite. Its pleural portions initially curve forwards abaxially, and then backwards in direction of posterolateral angles without reaching them (Figs 3a, b and 4a,b); the axial portion of the furrow is roughly transverse, almost reaching the posterior margin of the tergite sagittally, slightly departing from it adaxially. T2–10 are similar to T1, except for a progressive width reduction (tr.) and an increasing backward deflection of pleurae posteriorly, especially obvious in the holotype (Fig. 3a). They remain constant in length (sag.) until T7 or T8, and gently shorten further backwards. The morphology of the last two trunk tergites is essentially known from the holotype (Figs 3a and 4a, but see also Figs 3c and 4c). T11 is slightly longer (sag.) than T9 and T10, and apparently differs from all the tergites preceding it in having pleurae that sharply bend backwards abaxially, thus forming posteriorly-facing pleural spines (Figs 3b and 4b). T12 – the pretelsonic tergite – is preserved in oblique view (Fig. 3a,d,e), but most likely has a subtrapezoidal outline in dorsal/ventral view. It is roughly equal in length to the anteriormost trunk tergites, and therefore noticeably longer than T8–11. It is depressed medially into a broad shallow furrow, which is expressed as a ridge on the holotype counterpart (external mould mostly; Fig. 3d). Three elongate structures project from its posterior margin (Figs 3d,e and 4a) – they are straight, unsegmented, and narrow distally. Apparently none of them are complete distally, but one is slightly larger and c. three times longer than the other two. These other two spiniform structures have similar widths and lengths, and therefore likely represent a pair. These three structures are regarded as a telson and paired rami forming a furca, respectively.

**Figure 5:**
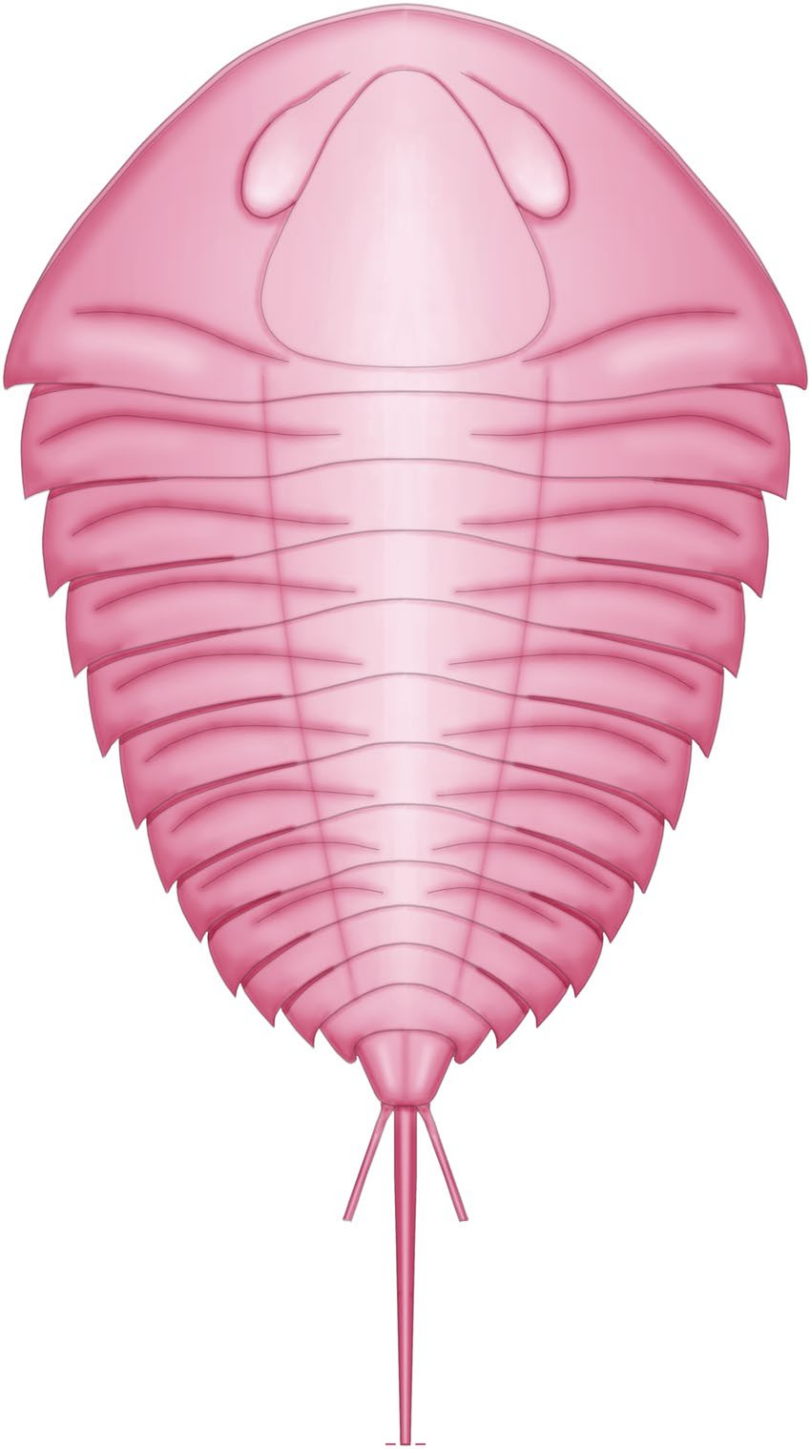
Morphological reconstruction of *Glypharthrus trispinicaudatus* sp. nov.

The dorsal exoskeletal surface is densely and evenly pitted along the entire body. A slight increase in pit diameter towards the axial region might represent the only spatial variation of sculpture (Fig. 3f). A reconstruction of the morphology of this new aglaspidid species is provided in Fig. 5.

### Phylogenetic analysis

To test the phylogenetic implications of these new observations, we scored them into a modified version of the character matrix of Ortega-Hernández *et al*.^22^, which incorporates recently described taxa and new characters (see Supplementary Note and Data). The results of the analysis of this dataset through parsimony and Bayesian inference are presented hereafter.

#### Parsimony analysis

**Figure 6:**
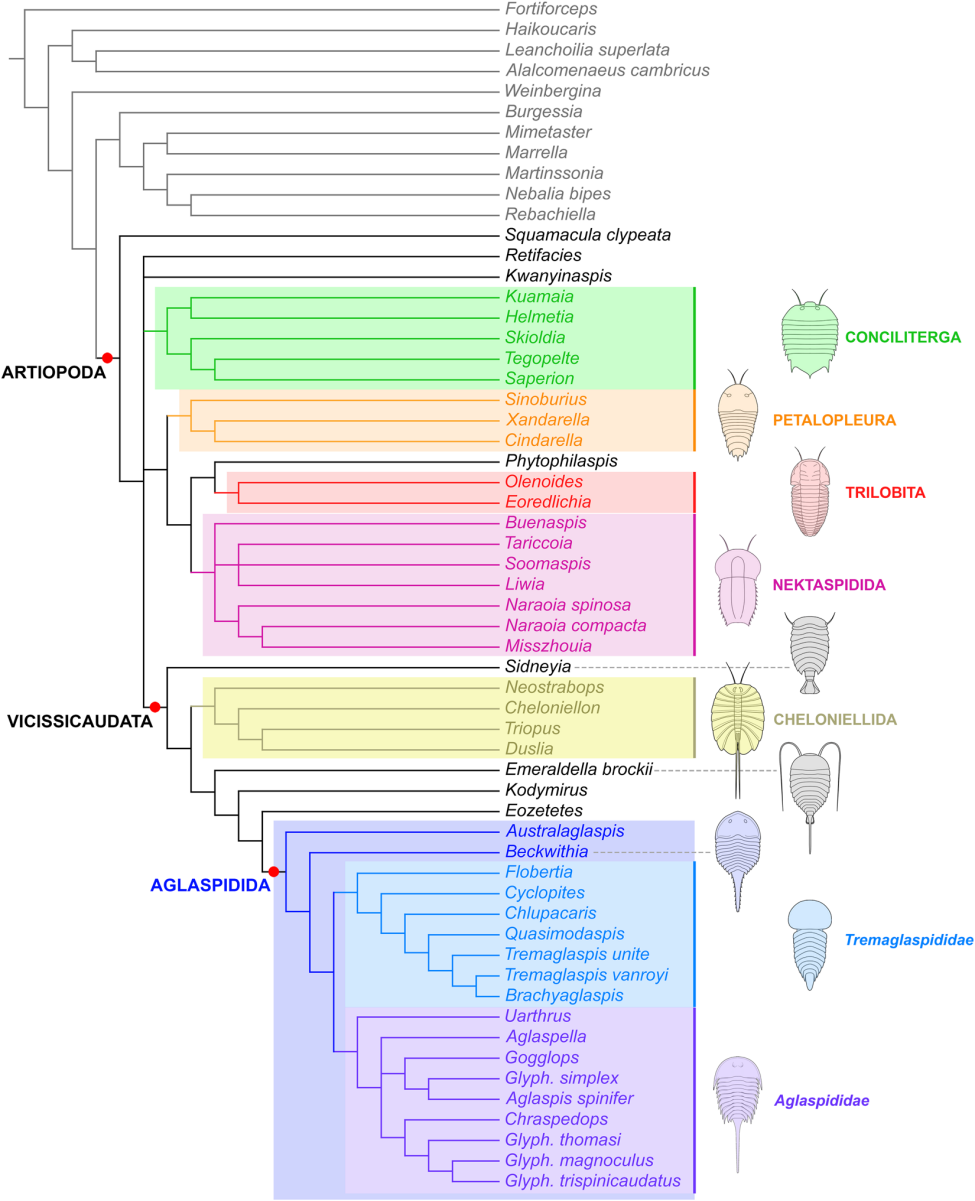
Strict consensus of six most parsimonious trees under equal weight (EW) parsimony. 252 steps, CI = 0.435, RI = 0.733. Character matrix available as Supplementary Data.

The EW-parsimony analysis produces six shortest trees (252 steps; CI = 0.435, RI = 0.733), the strict consensus of which is presented in Fig. 6. Artiopoda is recovered as a monophyletic clade in which *Squamacula* occupies the most basal phylogenetic position. The node above *Squamacula* comprises monophyletic Vicissicaudata, a grouping consisting of Petalopleura + [Nektaspidida + Trilobita], and Conciliterga. *Retifacies* and *Kwanyinaspis* occupy an unresolved position at this level. Although Trilobitomorpha is not resolved as a distinct clade, the analysis recovers monophyletic Conciliterga, Petalopleura, Nektaspidida, and the phosphatic biomineralized arthropod *Phytophylaspis pergamena* ^49^ as the sister-group to Trilobita.

Vicissicaudata comprises the Aglaspidida, Cheloniellida, *Emeraldella*, *Eozetetes*, *Kodymirus*, and *Sidneyia*. The node defining this clade is supported by the possession of a differentiated posterior trunk region (‘postabdomen’), which is 1) expressed dorsally as one, rarely two morphologically distinct tergites, 2) devoid of walking legs, and 3) bears a pair of pretelsonic appendicular derivatives. Interestingly, *Sidneyia* occupies the most basal position within this clade, while *Emeraldella* is recovered further up the tree, like *Kodymirus* and *Eozetetes*, as a basal member of the lineage leading to the Aglaspidida.

Aglaspidida is supported by the presence of a plate covering the anal region (post-ventral plate) and a biomineralized dorsal exoskeleton. It is essentially composed of two subgroups, the Aglaspididae Miller^50^ and the Tremaglaspididae fam. nov. (similar to the “Cambrian-type” and “Ordovician-type” groupings of Ortega-Hernández *et al*.^22^; see below), which are distinguished from one another by the presence of acute/spinose genal angles and a long spiniform tailspine (among other traits) in the Aglaspididae, and their absence in the Tremaglaspididae. *Australaglaspis* is not recovered within Aglaspididae despite having diagnostic characters of this group, which can be attributed to its poor preservation and uncertainty about its delicate morphology^6^. Likewise, *Beckwithia* was resolved within Aglaspidida but outside the two main subgroups. In this case, this is most likely the result of morphological differences between this taxon and other aglaspidids (e.g. absence of tergal processes, presence of axial spines in posterior trunk region). It is also worth noting that *Quasimodaspis*, a taxon previously regarded as close to, but definitely outside the Aglaspidida, is in our analysis recovered as a member of Tremaglaspididae fam. nov. Lastly, the new Chinese species, *Glypharthrus trispinicaudatus* sp. nov., forms a clade with two congeneric species and *Chraspedops* within the Aglaspididae.

#### Bayesian inference analysis

**Figure 7:**
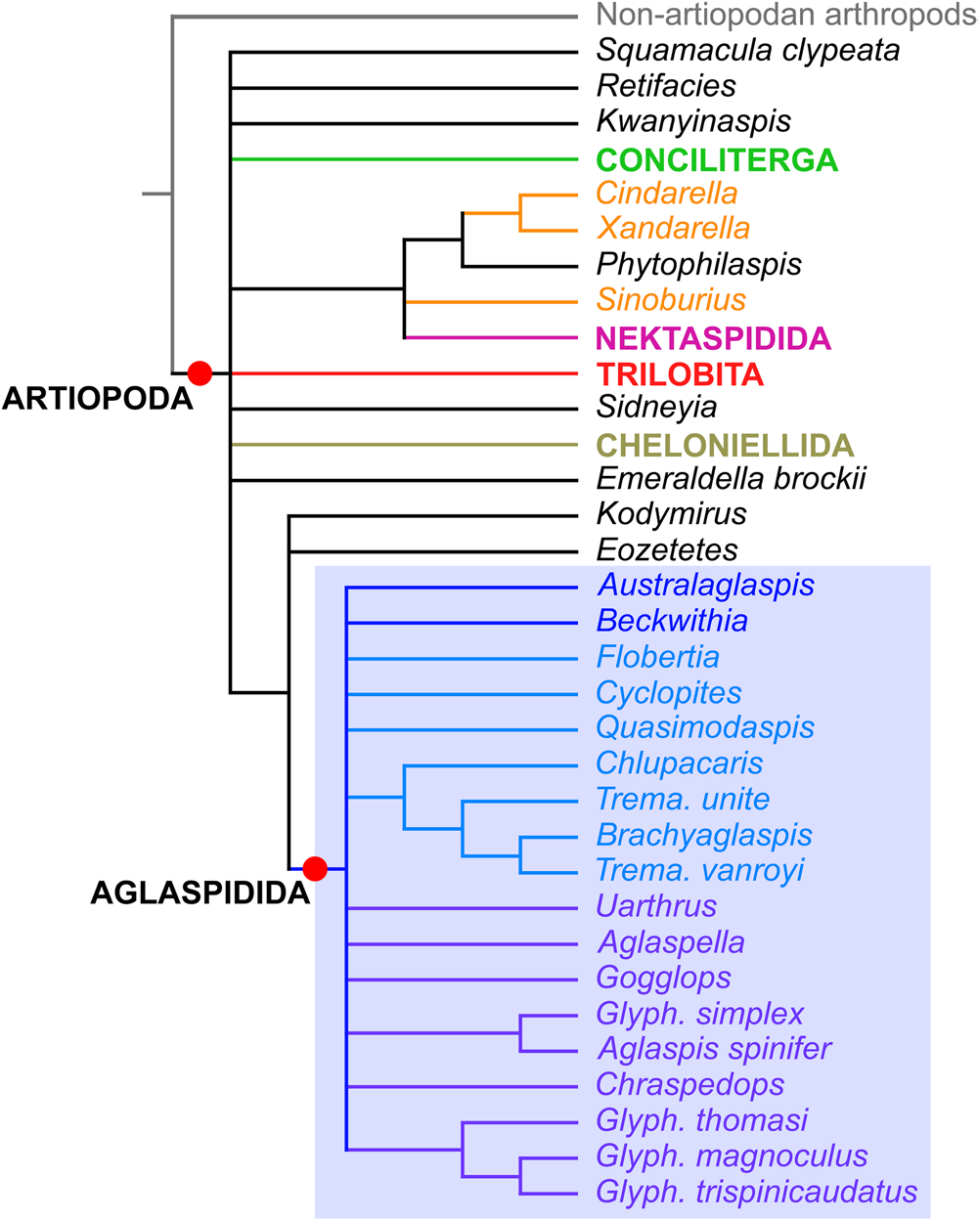
Consensus tree resulting from Bayesian analysis in MrBayes. Setting: Mk model, four runs, 20,000,000 generations, 1/1000 sampling resulting in 20,000 samples, 25% burn-in resulting in 15,000 samples retained. Relationships between non-artiopodan arthropods, and within Cheloniellida, Conciliterga, Nektaspidida, and Trilobita were resolved as in the EW-parsimony analysis (Fig. 6). Character matrix available as Supplementary Data.

The results of the Bayesian inference analysis are presented in Fig. 7. As expected from this methodology^51^, they offer considerably less tree resolution compared to those of the EW-parsimony analysis, but the basic structure of the tree remains largely identical, which attests to its robustness. Bayesian inference still resolves Artiopoda and within it, Aglaspidida, Cheloniellida, Conciliterga, Nektaspidida, and Trilobita as monophyletic clades. Internal relationships within all these artiopodan clades, except for Aglaspidida, are similar to those depicted by the EW-parsimony tree (Fig. 6). *Kwanyinaspis*, *Squamacula*, and *Retifacies* are retrieved outside all the aforementioned artiopodan clades. Among the groups traditionally regarded as trilobitomorphs, only the Petalopleura does not form a monophyletic taxon in this analysis, *Sinoburius* being retrieved in polytomy with the Nektaspidida and a clade (*Phytophilaspis* + (*Xandarella* + *Cindarella*)).

The greatest loss of tree resolution compared to EW-parsimony concerns the relationships between artiopodan main clades, with essentially no grouping into Trilobitomorpha or Vicissicaudata. The only exceptions are the association nektaspidids, petalopleurids and *Phytophilaspis* mentioned above, and a clade (*Eozetetes* + (*Kodymirus* + Aglaspidida)), which are both strongly reminiscent of the situation depicted by the EW-parsimony tree. The second major loss of resolution concerns the internal relationships within the clade Aglaspidida, which is otherwise composed of the exact same taxa (compare Figs 6 and 7). Some associations of taxa are still present, such as a group (*Chlupacaris* + (*Tremaglaspis unite* + (*Tremaglaspis vanroyi* + *Brachyaglaspis*))), but the clades Aglaspididae and Tremaglaspididae largely collapsed into a polytomy with *Australaglaspis* and *Beckwithia*. Interestingly, *Glypharthrus trispinicaudatus* sp. nov. is nested within a clade formed with two congeneric species (Fig. 7), as in the EW-parsimony analysis, which suggests that its phylogenetic position within the Aglaspidida is reliable.

#### Comparison with previous analyses

The results of the EW-parsimony analysis (Fig. 6) most closely resemble the topology obtained by Ortega-Hernández *et al*.^22^ (their Fig. 6b) under implied weights (k = 3). Both analyses recovered a monophyletic clade Artiopoda, including a well-defined Vicissicaudata comprising Aglaspidida, Cheloniellida, *Emeraldella*, and *Sydneyia*. The monophyletic sub-groups typically regarded as members of a clade Trilobitomorpha – namely Conciliterga, Nektaspidida, Petalopleura, and Trilobita – are also retrieved in both analyses, but the relationships between them differ markedly. In Ortega-Hernández *et al*.’s IW-parsimony analysis^22^ (their Fig. 6b), they do form a clade Trilobitomorpha, in which Petalopleura is a sister taxon to a clade [Nektaspidida + [Conciliterga + Trilobita]], while in our EW-parsimony analysis, Conciliterga is retrieved in polytomy with the Vicissicaudata and a group [Petalopleura + [Trilobita + Nektaspidida]]. This issue put aside, the internal relationships within each of these four clades are strictly similarly resolved whether they form a clade Trilobitomorpha^22^ or not (Fig. 6) – they are also in general agreement with other investigations of trilobitomorph phylogeny^19, 21, 23, 25, 31^. Otherwise, our analysis places *Squamacula* in a basal position within Artiopoda, as the sister-taxon of all the other members of the clade, while it was recovered as the sister-taxon of trilobitomorphs in Ortega-Hernández *et al*.^22^. The unresolved position of *Kwanyinaspis* relative to the main artiopodan clades (Fig. 6) is not surprising, considering that past analyses greatly disagreed about its placement within Artiopoda (i.e. sister to concilitergans^19, 21^; sister to aglaspidids^22^; unresolved within Trilobitomorpha^23^).

The internal topology within Vicissicaudata in our EW-parsimony tree (Fig. 6) also shows some notable differences compared to previous cladistic analyses. In the present study, *Sidneyia* is resolved as the most basal member of Vicissicaudata, whereas *Emeraldella* occupies the most basal position within the lineage that includes Aglaspidida. By contrast, Ortega-Hernández *et al*.^22^ (their Fig. 6b) resolved these taxa as part of a clade that also included Cheloniellida. Cotton and Braddy^25^ retrieved *Sidneyia* in a position similar to that depicted by our strict consensus tree (Fig. 6), but *Emeraldella* was recovered as the most basal of all vicissicaudatans (their ‘clade 5’). By contrast, Edgecombe *et al*.’s analysis^20^ resolved Cheloniellida as a sister taxon to a clade comprising *Emeraldella* and aglaspidids, similar to our EW-parsimony analysis, but this clade also included *Sidneyia*. The internal relationships within the Cheloniellida are again similarly depicted in Ortega-Hernández *et al*.’s analysis^22^ (their Fig. 6b), and our parsimony and Bayesian analyses (Figs 6 and 7).

Our EW-parsimony analysis refines the interrelationships within the clade that includes Aglaspidida compared to previous studies. *Emeraldella*, *Kodymirus*, and *Eozetetes* are resolved as the most basal members of this lineage, but outside of the Aglaspidida (Fig. 6; also Fig. 7 for *Kodymirus* and *Eozetetes*). The position of *Eozetetes* in this context is of particular interest, as Edgecombe *et al*.^24^ highlighted the morphological similarities of this taxon with both *Emeraldella* and aglaspidids (see also^2^). *Kodymirus* was also regarded as closely related to, but outside aglaspidids in ref. 22 (see also ref. 52). However, *Beckwithia* and *Quasimodaspis* were similarly positioned outside aglaspidids in their analysis, while they are retrieved within this clade in ours. Otherwise, the two analyses provide similar results regarding the presence of two main subgroups of aglaspidids; these were informally called ‘Cambrian-type’ and ‘Ordovician-type’ clades by Ortega-Hernández *et al*.^22^ and in subsequent studies^2, 5, 14^, and are formally defined as two separate families hereafter (see below). Internal relationships within each family differ in the two studies, but further comparisons are incompatible considering the differences in taxonomic diversity sampled in both analyses. The only major difference worth noticing is the inclusion of *Uarthrus* within the Aglaspididae in our study, whereas the position of this taxon within the Aglaspidida could not be resolved in ref. 22.

### Systematic palaeontology

The results of our phylogenetic analysis motivates the proposition of important changes to the systematics of the phylum Arthropoda.

Phylum Arthropoda von Siebold^53^

Subphylum Artiopoda (Hou and Bergström^54^)

#### Diagnosis

See ref. 26.

#### Remark

This taxon was originally described as a class^54^. We propose to elevate it to a subphylum level to reflect the widely accepted taxonomic ranks of some of its components (e.g. Class Trilobita) and the fact that these components form two to three major clades, including the Vicissicaudata, here regarded as superclasses.

Superclass Vicissicaudata (Ortega-Hernández, Legg and Braddy^22^) supercl. nov.

#### Diagnosis

Artiopods exhibiting the following unique combination of characters: trunk composed of 6 to 19 articulated segments (generally 10 to 12), all with wide (tr.) pleurae, except for the posteriormost one (exceptionally, the posteriomost two/three segments); posteriormost trunk segment bearing a pair of non-walking appendages, possibly secondarily lost in representatives in which this segment is fused to telson (e.g. some aglaspidids).

#### Remarks

The formal description of a superclass Vicissicaudata is motivated by the recognition of clear morphological characters that unites members of this clade, as supported by our phylogenetic analysis (Fig. 6). The authorship of the taxon is attributed to Ortega-Hernández, Legg and Braddy^22^, despite the absence of formal systematic description in their work, for we consider that the intention was present, as were the different components of a taxonomic description (i.e. ‘diagnosis’ and ‘composition’, see their p. 38; ‘etymology’, see their p. 37).

Order Aglaspidida Walcott^33^

#### Emended diagnosis

Vicissicaudatans exhibiting the following unique combination of characters: cuticle primarily lightly biomineralized (phosphatic); cephalon devoid of ecdysial sutures dorsally; dorsal eyes sessile, merging with rest of cephalon anteromedially, typically located in far-anterior position and abutting triangular glabellar region (when present); may be lost in derived taxa; four, possibly five pairs of cephalic appendages present; trunk tergites freely articulating, except for posteriormost one that is often fused to telson; trunk pleurae commonly carrying a pair of anterior tergal processes; bipartite postventral plate located beneath posteriormost one or two trunk tergites and when present, base of tailspine.

#### Genera included

Family Aglaspididae: *Aglaspis* Hall^55^ (type genus), *Aglaspella* Raasch^7^, *Aglaspoides* Raasch^7^, *Chraspedops* Raasch^7^, *Glypharthrus* Raasch^7^, *Gogglops* Siveter *et al*.^16^, *Hesselbonia* Lerosey-Aubril *et al*.^9^, *Setaspis* Raasch^7^, *Tuboculops* Hesselbo^8^, *Uarthrus* Raasch^7^; Family Tremaglaspididae fam. nov.: *Brachyaglaspis* Ortega-Hernández *et al*.^14^, *Chlupacaris* Van Roy^17^, *Cyclopites* Raasch^7^, *Flobertia* Hesselbo^8^, *Quasimodaspis* Waggoner^56^, *Tremaglaspis* Fortey and Rushton^11^; Not assigned to a particular family: *Australaglaspis* ^6^, *Beckwithia* Resser^57^. Tentatively included: *Angarocaris* Černyšev^58^, *Chacharejocaris* Černyšev^59^, *Girardevia* Andreeva^60^, *Obrutschewia* Černyšev^59^, *Rozhkovocaris* Rosov^15^.

Family Aglaspididae Miller^50^

#### Emended diagnosis

Aglaspidids exhibiting the following unique combination of characters: body flat, with wide (tr.) pleural regions; cephalon with acute to spinose genal angles, prominent eyes, and commonly bearing furrows; trunk ancestrally composed of twelve articulated tergites, and a spiniform telson that is commonly fused with T12 to form a long tailspine with broad (tr.) base in more derived forms.

#### Genera included

See list of genera included within the order above.

#### Remarks

*Australaglaspis* most likely belongs to this family, for it displays most of its diagnostic characters (e.g. prominent eyes, acute genal angles, eleven trunk tergites and a long tailspine with broad base). Yet, it was recovered in a more basal position within the Aglaspidida in our EW-parsimony analysis (Fig. 6), most likely due to a limited understanding of some aspects of the morphology of this taxon (e.g. presence or absence of tergal processes), itself a direct consequence of the poor preservation of the material found in the Paibian strata of Tasmania. Awaiting for the discovery of more, possibly better preserved specimens from these deposits, this aglaspidid genus is not assigned to a particular family. This is also the case of *Beckwithia* from the Guzhangian Weeks Formation of Utah, USA^57, 61^, which exhibits several characteristics of the Aglaspididae, such as a flat body with wide (tr.) pleural regions, a cephalon with prominent eyes, genal spines, and sometimes shallow furrows, and a tailspine with a broad base. However, ongoing re-investigation of this taxon by two of us (RLA, JOH) shows that it differs from all the other aglaspidids regarding the morphology of its tailspine and the presence of a single row of median spines on the posterior trunk region. Consequently, we are reluctant to assign *Beckwithia* to the family Aglaspididae in the present contribution.

Genus *Glypharthrus* Raasch^7^

#### Type species

*Glypharthrus thomasi* (Walter^62^) from the Lodi Member of the Saint Lawrence Formation, Furongian (Jiangshanian), Wisconsin and Iowa, USA.

#### Species included

*Glypharthrus thomasi* (Walter^62^); *G. simplex* (Raasch^7^) from the Lodi Member of the Saint Lawrence Formation, Furongian (Jiangshanian), Wisconsin and Iowa, USA; *G. vulpes* Raasch^7^ from the Ironton Member of the Wonewoc Formation, Furongian (Paibian?–Jiangshanian), Wisconsin, and possibly the Deadwood Formation (=*G. deadwoodensis* of ref. 7), Furongian (Paibian?–Jiangshanian; *Elvinia* trilobite Zone), South Dakota, USA; *G. magnoculus* Lerosey-Aubril *et al*.^2^ from the lower part of the McKay Group, Furongian (Jiangshanian), British Columbia, Canada; *G. trispinicaudatus* sp. nov. from the Sandu Formation, Furongian (Jiangshanian), Guangxi, China.

#### Emended diagnosis

Genus of Aglaspididae characterized by the following unique combination of characters: cephalon with particularly narrow, elevated, anterolateral marginal rim, and posterior furrows defining wide (exs.) posterior border abaxially; eleven to twelve trunk tergites, with T1–10 (at least) bearing pleural furrows, T11 long (sag.), and T12 (when present) even longer (sag.), trapezoidal in outline, and bearing a pair of furcal rami.

#### Remarks

We follow Hesselbo^8^ and Lerosey-Aubril *et al*.^2^ in regarding *Glypharthrus deadwoodensis* Raasch^7^ as a junior synonym of *G. vulpes* ^7^, and *Aglaspoides* and *Glypharthrus* as distinct genera (*contra* ^22^). The above diagnosis is essentially that of Lerosey-Aubril *et al*.^2^, except for the addition of a new character: the presence of a pair of furcal rami born by T12. These structures have as-yet been observed in a single specimen of *G. trispinicaudatus* sp. nov. only (Fig. 3a,d,e). However, we believe that they might have originally occurred in more, if not all representatives of the genus, but like the other appendages they were too delicate to be preserved. This seems particularly likely with *G. magnoculus*, in which the presence of a distinct, long T12 has been clearly established, and which is only known from a single specimen that does not preserve any remains of appendages^2^. Actually, the preservation of aglaspidid appendages is exceedingly rare, having been reported in only two specimens to date, but none belonging to a species of *Glypharthrus* ^7, 8, 63^. If we are correct in regarding the furcal rami of *G. trispinicaudatus* sp. nov. as modified appendages, they were probably as prone to decay as the other appendages, and therefore rarely fossilized. In *G. simplex*, *G. thomasi*, and *G. vulpes*, another explanation for their absence might relate to the evolution of posterior trunk morphology. Indeed, it is not clear in these three taxa whether the post-cephalic region comprises twelve trunk segments and a narrow spiniform telson, as in *G. magnoculus* and *G. trispinicaudatus* sp. nov., or eleven trunk segments and an anteriorly enlarged tailspine, as in most Aglaspididae. In the latter case, the evolution of the tailspine by fusion of the telson and T12^2, 17^ might have resulted in the loss of the furcal rami born by the twelfth trunk segment. Lastly, it cannot be entirely ruled out that the furcal rami evolved into the postventral plate (see discussion below), especially since they were not observed in association with this plate in *G. trispinicaudatus* sp. nov. This could explain the absence of furcal rami in *G. simplex*, a species with a well-developed postventral plate^8^.

*Glypharthrus trispinicaudatus* sp. nov. Figures 3–5

#### Etymology

From the Latin *‘tri’*, ‘*spina*’ and ‘*cauda’*, meaning ‘three’, ‘spines’ and ‘tail’, respectively, in reference to the furcal rami and spiniform telson projecting from the posterior end of the body.

#### Diagnosis

Species of *Glypharthrus* characterized by the following unique combination of characters: cephalon semi-elliptical to semi-circular in outline, with particularly narrow (exs.) preocular field, long (exs.), rather well-defined glabellar region, and narrow (tr.) elongate eyes; trunk tergites long (sag.), essentially rectangular anteriorly, with all but T12 bearing shallow pleural furrows extending to the axial region adaxially.

#### Material, locality, horizon

NIGPAS 165042 (Figs 3a,b,d,e and 4a,b), holotype, part and counterpart of complete, dorsoventrally flattened dorsal exoskeleton associated with a pair of furcal rami; NIGPAS 165043 (Figs 3c,f and 4c), paratype, partial cephalon associated with the remains of eleven, slightly disarticulated trunk tergites; NIGPAS 165044 (Figs 3g–i and 4d,e), paratype, part and counterpart of a partial dorsal exoskeleton composed of the cephalon and eight trunk tergites; calcareous mudstone of the Sandu Formation, *Probinacunaspis nasalis-Peichiashania hunanensis* trilobite Zone, Jiangshanian, Furongian, Guole area, Jingxi County, Guangxi Province, China.

#### Description

See description above.

#### Remarks

*Glypharthrus trispinicaudatus* sp. nov. and *G. simplex* are unique amongst the representatives of the genus in having small eyes and long (sag., exs.), rectangular anterior trunk tergites. However, the eyes are particularly elongate (exs.) in the Chinese species, which also differs from *G. simplex* by its much narrower (exs.) preocular field and its better-defined, possibly more robust glabellar region. The last two characters also permit to distinguish the new taxon from *G. thomasi*, a species with a slender glabellar region and large eyes. Additionally, *G. trispinicaudatus* exhibits a semi-elliptical to semi-circular cephalic outline, while it is semi-parabolic in *G. thomasi*. *Glypharthrus vulpes* is almost exclusively known from fragmentary cephala, but these cephala display large eyes and spinose genal angles, two characters absent in the new species. Lastly, *G. trispinicaudatus* sp. nov. and *G. magnoculus* both possess a trapezoidal twelfth trunk tergite, but these species are otherwise easy to distinguish. Indeed, the Chinese species lacks the huge eyes, the deeply-incised furrows, and the short triangular trunk characterizing the recently described Canadian taxon.

Family Tremaglaspididae fam. nov.

#### Diagnosis

Aglaspidids exhibiting the following unique combination of characters: body typically strongly vaulted; cephalon with rounded genal angles, reduced to no dorsal eyes, and no furrows; trunk composed of eleven or fewer tergites (minimum six), usually with axial region wider (tr.) than pleural regions; tailspine typically shorter than half body length (sag.; tailspine excluded).

*Genera included*. See list of genera included within the order above.

#### Remarks

The family Tremaglaspididae fam. nov. essentially corresponds to the ‘Ordovician-type aglaspidids’ of Ortega-Hernández *et al*.^22^ and subsequent workers^2, 5, 14^. Since its first recognition, this clade has gained support from the observation of a similar set of characters in two additional taxa, namely *Brachyaglaspis singularis* Ortega-Hernández *et al*.^14^ and *Tremaglaspis vanroyi* Lerosey-Aubril *et al*.^5^. The latter species also demonstrated that tremaglaspidids were already separated from other Aglaspidida by the end of the Guzhangian. Despite surviving until the Katian age and therefore spanning c. 50 Myr, this family apparently never reached a diversity peak comparable to the one reached by the Aglaspididae during the Jiangshanian age. *Longquania bispinosa* Luo and Hou (in ref. 64) from the Cambrian Stage 4 Guanshan Biota of South China is somewhat similar to tremaglaspidids in general morphology (e.g. a large rounded cephalon; see ref. 65), but also exhibits features never observed in this family (e.g. 12 trunk tergites) or in the Aglaspidida as a whole (e.g. trunk tergites with raised axial region bearing paired axial nodes, a short bifurcate tailspine). Also, it seems to us that the inclusion of this species within the Tremaglaspididae fam. nov. or even within the Aglaspidida is not justified at this stage, although we acknowledge that discovery of new specimens or a thorough restudy of the two fragmentary specimens yet available might prove us wrong.

## Discussion

### Evolution of trunk tagmosis in aglaspidids

**Figure 8:**
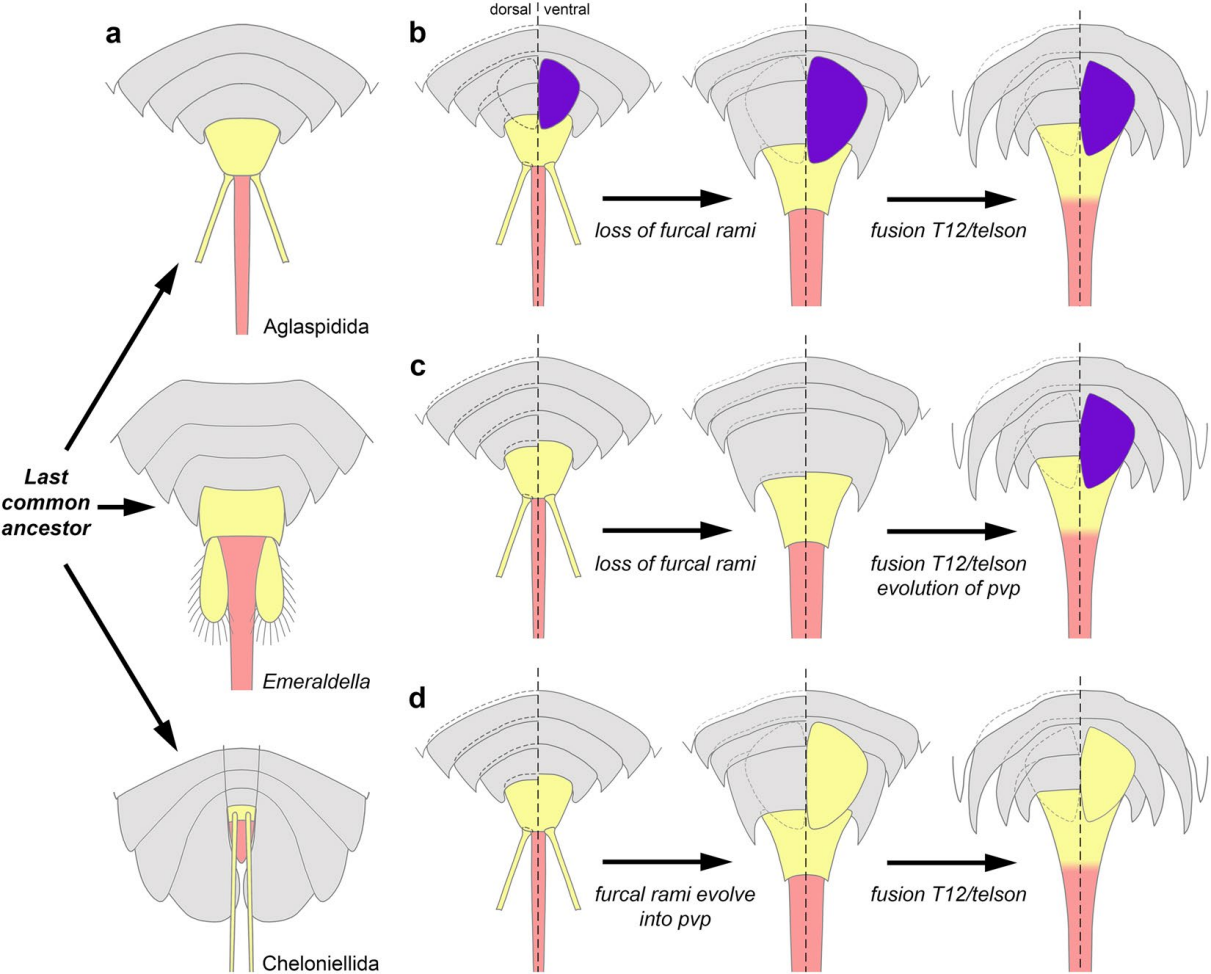
Morphology and evolution of the posterior trunk region within the Vicissicaudata. (**a**) morphology of the posterior trunk regions of aglaspidids (ancestral condition), *Emeraldella*, and cheloniellids; a morphologically-distinct, pretelsonic segment bearing appendage derivatives (in yellow) occurs in front of the telson (in pink) in all these taxa. (**b**–**d**) evolutionary scenarios describing the fusion of the pretelsonic segment and telson into a tailspine, and the relationship between pretelsonic appendages (furcal rami) and postventral plate (pvp) in aglaspidids. (**b**) aglaspidid ancestor possessed pretelsonic appendages (in yellow) and postventral plate (in purple), but the former structures disappeared in most descendants. (**c**) aglaspidid ancestor possessed pretelsonic appendages only (in yellow), which were lost in most descendants – the postventral plate (in purple) evolved independently. (**d**) aglaspidid ancestor possessed pretelsonic appendages only, which later evolved into the postventral plate in most descendants (both structures are in yellow).

The discovery of *G. trispinicaudatus* sp. nov. illuminates the early evolution of trunk tagmosis in aglaspidids. Indeed, this new taxon further illustrates the association of a twelfth pretelsonic-like segment and a simple terminal spine (telson) in a representative of this group^2^, and thus directly supports the hypothesis put forward by Van Roy^17^. More importantly, it shows that this additional trunk segment not only differs from more anterior ones in a similar way than the pretelsonic segment of other vicissicaudatans, but it may also bear a pair of modified appendages (i.e. the furcal rami; Fig. 8a). This is a characteristic of the non-aglaspidid vicissicaudatan pretelsonic segment, although the associated appendages greatly differ in morphology from one taxon to another: short to extremely long, dorsally-inserted furcal rami in cheloniellids; short, ovoid caudal flaps in *Emeraldella*; and large, triangular uropods in *Sidneyia*. The postventral plate(s) – a bipartite ventral sclerite of possible appendicular origin diagnostic of the order Aglaspidida – was regarded as homologous to the pretelsonic appendages of other vicissicaudatans by some workers^17, 25^, but not by all. For instance, Edgecombe *et al*.^20^ considered that the dorsal insertion of cheloniellid furcal rami prevents from regarding them as homologous to the postventral plate(s) of aglaspidids or the caudal flaps of *Emeraldella*. On the other hand, Ortega-Hernández *et al*.^22^ questioned that a pretelsonic segment was fused to the telson in aglaspidids; instead, they regarded the unique morphology of aglaspidid postventral plate(s) as incompatible with the hypothesis of it (/them) being homologous to the pretelsonic appendages of other vicissicaudatans. A postventral plate(s) was not observed in any of the three specimens of *G. trispinicaudatus* sp. nov., but the limited amount of material available so far does not permit to definitely exclude that it was present in this taxon. As a consequence, it is not possible to determine whether the furcal rami and postventral plate(s) are totally distinct structures (Fig. 8b,c) or if the postventral plate(s) evolved from the former (Fig. 8d). In any case, it is now reasonable to assume that the common ancestor of all aglaspidids was similar to non-aglaspidid vicissicaudatans in possessing a morphologically differentiated last trunk segment, the pretelsonic segment, which bore a pair of modified appendages instead of walking legs.

### New phylogenetically significant characters

The high degree of resolution within Vicissicaudata obtained with EW-parsimony stresses the significance of the new taxa and characters incorporated into the matrix of Ortega-Hernández *et al*.^22^. The re-interpretation of the morphology of the posterior trunk region (‘postabdomen’) allowed a refined coding of this aspect of artiopodan morphology. The resulting three characters (morphological differentiation of a ‘postabdomen’; presence/absence of ‘postabdominal’ walking legs; presence/absence of ‘postabdominal’ modified appendages) support the clade Vicissicaudata in our EW-parsimony analysis, and are therefore regarded as diagnostic above.

**Figure 9:**
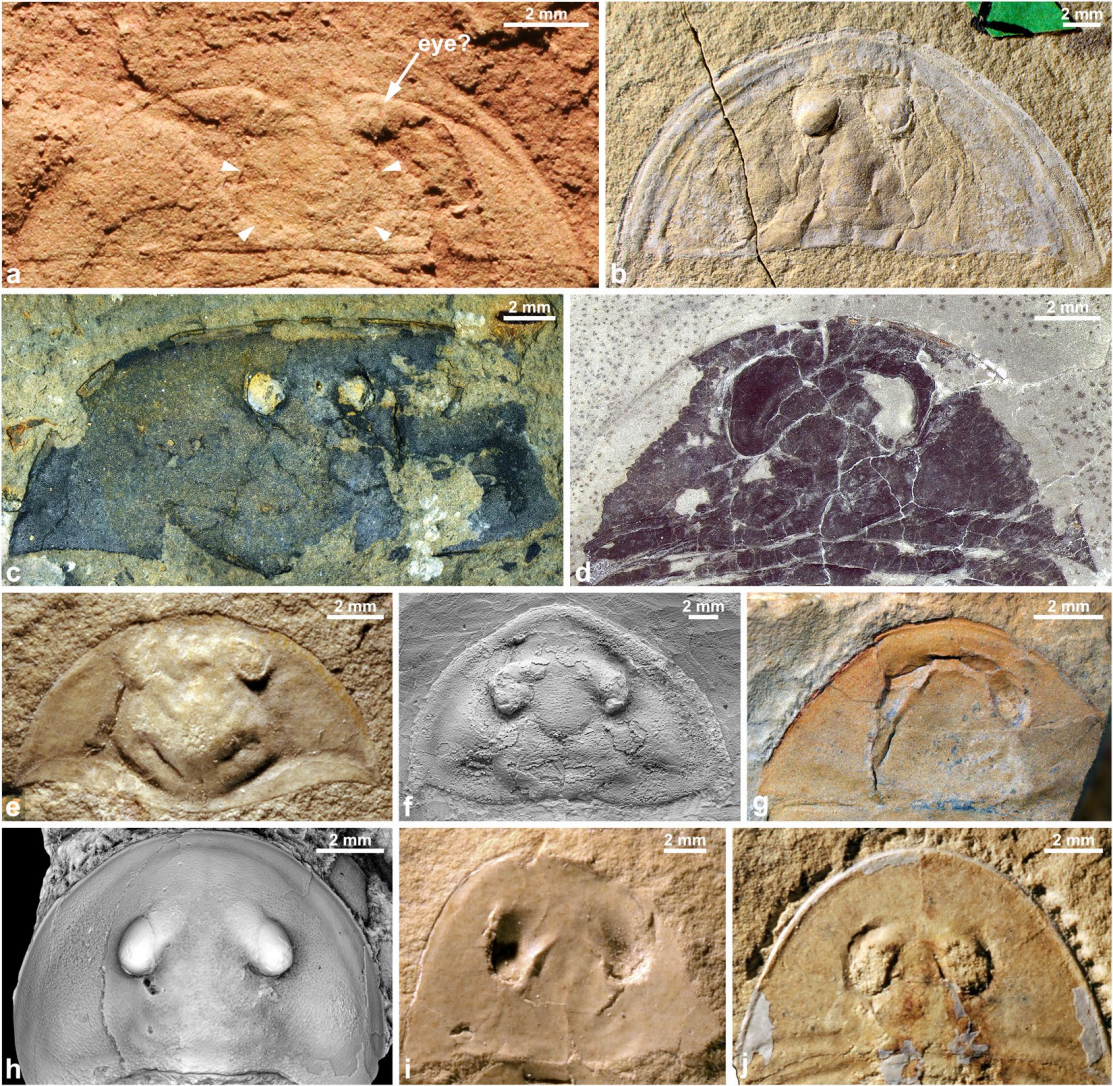
Diversity of shape, size, and location of the eyes in aglaspidids and *Eozetetes*. The eyes abut the typically triangular glabellar region anteriorly, and gradually merge with the rest of the cephalon anteromedially; when occupying a more posterior position, they are prolonged anteriomedially by massive ridges. A, *Eozetetes gemelli* (SAM P48369a – Cambrian Series 2, Stage 4; Emu Bay Sh., Australia), reinterpreted as showing an eye abutting a triangular glabellar region (arrow heads) anteriorly, courtesy of J. Paterson; B, *Aglaspis spinifer* (USNM 98912 – Furongian, Jiangshanian; St Lawrence Formation, USA); C, *Aglaspella sanduensis* (NIGPAS 157028 – Furongian, Jiangshanian; Sandu Formation, China); D, *Glypharthrus magnoculus* (UA 14332 – Furongian, Jiangshanian; McKay Gr., Canada); E, *Chraspedops modesta* (MPM no number – Furongian, Jiangshanian; St Lawrence Formation, USA); F, *Beckwithia typa* (BPM 1060 – Cambrian Series 3, Guzhangian; Weeks Formation, USA); G, *Glypharthrus trispinicaudatus* (NIGPAS 165042 – Furongian, Jiangshanian; Sandu Formation, China); H, *Gogglops ensifera* (NIGPAS 160539 – Upper Ordovician, Sandbian; Yaoxian Formation, China); I, *Aglaspoides sculptilis* (MPM 11168 – Furongian, Jiangshanian; St Lawrence Formation, USA); J, *Glypharthrus thomasi* (MPM 11163 – Furongian, Jiangshanian; St Lawrence Formation, USA).

The inclusion of new characters applicable to the dorsal eyes also contributes towards a better resolution of the relationships within vicissicaudatan taxa (Characters 82–84 in Supplementary Note and Data). These characters were introduced to depict particularities of the eyes of aglaspidids: 1) their typical far-forward location on the cephalon; 2) the fact that they consistently abut the glabellar region (when this region is visible); and 3) the fact that they merge with the rest of the cephalon anteriomedially. Figure 9 illustrates the persistence of these characters in aglaspidids and possibly *Eozetetes*, despite a great diversity of shapes, sizes, and location of the eyes. The distribution of these three characters within the EW-parsimony strict consensus tree confirms that they are restricted to aglaspidids and closely related taxa, and therefore are diagnostic for this lineage. Aglaspidida share the far-forward location of the eyes and their position abutting the glabellar region with *Kodymirus* and possibly *Eozetetes* (compare Fig. 9a herein and ref. 24 for alternative interpretations of this taxon’s cephalic morphology), whereas the merging of the eyes with the cephalon anteriomedially is a character apparently unique to the Aglaspidida. Indeed, this character is expressed in all aglaspidid representatives with dorsal eyes that are known from sufficiently well-preserved material (i.e. coded as uncertain in *Australaglaspis*, *Flobertia*, and *Chlupacaris*, and non-applicable in aglaspidids lacking dorsal eyes). Character mapping indicates that the position of the eyes on the head, and their relationship with the glabella, support the grouping of *Kodymirus* and *Eozetetes* with aglaspidids, despite the collapse of Vicissicaudata in the Bayesian analysis. Given the distinctiveness of these ocular characters, their inclusion with the diagnosis of the order seems appropriate (see above). In this context, the anteromedian merging of the eyes with the rest of the cephalon is of particular significance (Fig. 9b–j), for it could be used as a diagnostic feature of Aglaspidida in the absence of information pertaining to the presence of a postventral plate, as when this plate is not preserved or not observable (i.e. concealed under the dorsal exoskeleton).

### Diversification dynamics of early artiopods

The late Cambrian has been repeatedly described as a time of diversity plateau^66–68^ (see ref. 69 for a different view), which might lead to the misconception that it was a relatively uneventful period in the history of life. In fact, the time interval between the Cambrian Explosion and the GOBE was truly pivotal in the evolution of the biosphere. The plateau described by palaeodiversity curves masks the onset of a profound restructuring of marine communities: the decline of the Cambrian Evolutionary Fauna commenced, but it was almost perfectly compensated by the rise of the Palaeozoic Evolutionary Fauna^70^. Meanwhile, marine phytoplankton diversity practically doubled^71^, and arthropods started venturing onto land (e.g. ref. 72). As illustrated by aglaspidids, the late Cambrian was also a time of morphological and ecological innovations, which saw the evolution of numerous new components of marine ecosystems. Some of these newcomers would become dominant in the Ordovician as parts of the Palaeozoic Evolutionary Fauna^73^, whereas others would have a more limited evolutionary success (e.g. refs 74 and 75).

In this regard, the confrontation of the palaeodiversity and phylogenetic patterns of artiopods is particularly interesting (Fig. 1), for it allows drawing a parallel between the split of this branch of the arthropod tree into Trilobitomorpha and Vicissicaudata (or Conciliterga, non-concilitergan trilobitomorphs, and Vicissicaudata, as in our analysis) on the one hand, and the separation in time of the initial diversifications of these two (/three) groups on the other. This observation suggests that the early history of this group comprises two distinct diversification pulses, which somewhat recalls the general situation depicted for marine invertebrates during the Early Palaeozoic. However, these two pulses are only separated by a c. 20 Myr interval, which is about twice less than the time interval between the Cambrian Explosion and the GOBE. A partial explanation for this unusual pattern might come from the fact that the initial (and main) diversification of vicissicaudatans is essentially that of aglaspidids, a group that preferentially inhabited relatively shallow-water environments^5^). The greater instability of such environments is thought to cause increased competition between species, which would ultimately nurture morphological and ecological innovations (e.g. refs 76 and 77). The precocious second diversification pulse of artiopods would thus be related to the fact that it essentially occurred in such unstable, shallow-water environments.

Testing this assumption is beyond the scope of the present work, but these considerations illustrate how complementing the scarce fossil record of non- or weakly biomineralizing metazoans living during the late Cambrian might ultimately contribute to an enhanced understanding of the early diversification of animals as a whole. More generally, developing the study of the few Konservat-Lagerstätten known from the time interval between the Cambrian Explosion and the GOBE, such as the Guzhangian Weeks Formation^78^, the Jiangshanian Sandu Formation^34^ and McKay Group^2^, and of course the Lower Ordovician Fezouata Shale^79, 80^, is more than ever critical.

## Material and Methods

The specimens were photographed dry or immersed in dilute ethanol using a Leica DFC420 digital camera mounted on a Leica MZ16 microscope. Series of images were taken with manual focusing at different focal planes, and subsequently stacked and assembled in Adobe Photoshop CS6 to create high-resolution composites. These photographs and the same software were used to make interpretative drawings and a reconstruction. EDS analyses were performed using a LEO1530VP scanning electron microscope (environmental mode) combined with an Energy-Dispersive X-ray (EDX) module OXFORD INCA300. The specimens are deposited in the collections of the Nanjing Institute of Geology and Palaeontology, Chinese Academy of Sciences (NIGPAS 165042–165044), Nanjing, China. Other institutional abbreviations (Fig. 9): BPM, Back to the Past Museum (Mexico); MPM, Milwaukee Public Museum (USA); SAM, South Australian Museum (Australia); UA, University of Alberta (Canada); USNM, National Museum of Natural History (USA).

Phylogenetic analyses were conducted using an updated version of the character matrix designed by Ortega-Hernández *et al*.^22^, which consists of 58 taxa and 86 characters (see Supplementary Note and Data). The dataset was analysed through parsimony and Bayesian inference to compare the results from these different methodologies (e.g. refs 50 and 81). The parsimony analysis was run in TNT^82^ under New Technology Search, using Driven Search with Sectorial Search, Ratchet, Drift, and Tree fusing options activated in standard settings under equal weights (EW)^83, 84^. The analysis was set to find the minimum tree length 100 times and to collapse trees after each search. All characters were treated as unordered. The Bayesian analysis was performed in MrBayes using the Monte Carlo Markov-chain (Mk) model for morphology^85^ for 20 million generations (four chains), with every 1000^th^ sample stored (resulting in 20,000 samples), and 25% burn-in (resulting in 15,000 retained samples).

## Electronic supplementary material

Supplementary Information

Supplementary Data
