# Supplementary material for: The Vicissicaudata revisited – insights from a new aglaspidid arthropod with caudal appendages from the Furongian of China
Source: Sci Rep. 2017 Sep 11;7:11117. doi: 10.1038/s41598-017-11610-5 (PMC5593897; doi:10.1038/s41598-017-11610-5)
Supplement: Supplementary file 1 — Supplementary Information [file 41598_2017_11610_MOESM1_ESM.pdf]

Lerosey-Aubril, Zhu and Ortega-Hernández. The Vicissicaudata revisited – insights from a new aglaspidid arthropod with caudal appendages from the Furongian of China

Supplementary Note – Modifications to dataset of Ortega-Hernández et al.'s (2013)

We employed an updated version of the character dataset by Ortega-Hernández et al. (2013) to analyse the phylogeny of Artiopoda, with particular emphasis on the interrelationships within Aglaspidida and other closely related forms (dataset available as Supplementary Data online).

1. Taxa considered

The following taxa were added to the dataset: *Brachyaglaspis singularis* Ortega-Hernández et al., 2016, *Eozetetes gemmeli* Edgecombe et al., 2017, *Glypharthrus magnoculus* Lerosey-Aubril et al., 2017, *Glypharthrus trispinicaudatus* sp. nov., *Gogglops ensifera* Siveter et al., in press, *Phytophilaspis pergamena* Ivanstov, 2009, and *Tremaglaspis vanroyi* Lerosey-Aubril et al., 2013.

The following taxa were excluded: *Dicranocaris,* Eurypterida, *Jianfengia*, *Nettapezoura*, *Pseudoarthron*, Pycnogonida, *Yohoia*.

2. Characters

Most characters were employed unchanged, albeit with some coding corrections following comments by Stein et al. (2013). Details relevant to modified and new characters are given below. Note that character 69 from Ortega-Hernández et al. (2013) is now effectively integrated into Character 60, and thus the numbering of the Character 69 to 81 depicted below would correspond to character 70 to 82 *sensu* Ortega-Hernández et al. (2013).

2.1. Modified characters

Char. 60. Postabdomen morphologically differentiated relative to thoracic tergites into preterminal tergite

(0) absent

(1) present – preterminal tergite free (e.g. some aglaspidids, cheloniellids, Emeraldella, Sidneyia*)*

(2) present – preterminal tergite fused with telson (e.g. most aglaspidids)

(-) inapplicable: pygidium present

Remarks. This character has been modified from Char. 60 in Ortega-Hernández et al. (2013), to incorporate recent observations on the morphology of aglaspidids that support the homology of the anterior expansion of the tailspine of some aglaspidids with the pretelsonic segment (12th trunk segment) of others (see Lerosey-Aubril et al., 2017). This character is scored as absent in crustaceomorphs as the abdomen is defined by the lack of appendages, but not by morphologically distinct posterior tergites.

Char. 73. Ventral sclerotized plate covering anal region

(0) absent

(1) present (e.g. Aglaspidida, Sidneyia)

Remarks. This character has been modified from Char. 74 in Ortega-Hernández et al. (2013) to reflect the fact that both the bipartite postventral plate of aglaspidids (e.g. Heselbo, 1992; Van Roy, 2006; Fortey and Rushton, 2009; Ortega-Hernández et al., 2016) and the anal plate of *Sidneyia* (e.g. Bruton, 1981; Zacaï et al., 2016) are similar in shape and location (see also Lerosey-Aubril et al., 2017).

2.2. New characters

Character 82. Dorsal eyes confined to anterior half of cephalon

(0) absent (e.g. Trilobita)

(1) present (e.g. Aglaspidida)

(-) inapplicable: dorsal sessile eyes absent

Remarks. This new character reflect the organization observed in several aglaspidids, in which the location of dorsal eyes seems essentially restricted to the anterior part of the cephalon (Fig. 9), in contrast to the more variable location of the eyes of trilobites for instance. This character is scored as present in *Eozetetes* (*contra* Edgecombe et al. 2017) as we consider that the location of the paired sediment bulges in the holotype result from the presence of eyes on the dorsal exoskeleton.

Char. 83. Dorsal eyes abutting glabella anteriorly

(0) absent (e.g. Trilobita)

(1) present (e.g. Aglaspidida)

(-) inapplicable: glabellar region not differentiated, or dorsal eyes are absent

Remarks. This new character reflects the close anatomical relationship observed between the eyes and the glabellar region in Aglaspidida. Even when located more posteriorly, the eyes abut the glabellar region and they form anterior extensions (‘ocular ridges’) connecting them to areas located bordering the glabellar region anteriomedially. This is in striking contrast to the situation in other artiopods, especially trilobites, where the eyes are frequently located far from the glabella abaxially.

Char. 84. Dorsal eyes progressively merge with rest of cephalon anteriorly

(0) absent (e.g. Trilobita)

(1) present and eyes are separate (e.g. Glypharthrus)

(2) present, and eyes are medially fused (e.g. Cyclopites)

(-) inapplicable: glabellar region not differentiated, or dorsal eyes are absent

Remarks. This new character relates to a morphological particularity of aglaspidid eye. It is separated from the rest of the cephalon by a sharp break in slope or a furrow all around, except anteriomedially. There, it progressively decreases in relief to merge with the rest of the cephalon. This is true whether the eye is prolonged anteriomedially by an 'ocular ridge' or not (Fig. 9).

Char. 85. Nature of the ventral sclerotized plate covering the anal region

(0) anal plate (e.g. Sidneyia*)*

(1) postventral plate (e.g. Aglaspidida)

(-) inapplicable: ventral sclerotized plate covering anal region absent

Remarks. This new character distinguishes between the possibly morphological modification of the ventral sclerite, either as the anal plate of *Sidneyia* or the aglaspidid postventral plate.

Char. 86. Nature of differentiated preterminal tergite

(0) tergite with reduced pleurae (e.g. Weinbergina)

(1) cylindrical tergite without pleurae (e.g. Aglaspidida, Cheloniellida, Emeraldella)

(-) inapplicable: postabdomen not differentiated into preterminal tergite

Remarks. The differentiated preterminal tergite may be expressed simply by the presence of reduced pleurae, as in *Weinbergina*, or as a distinctive cylindrical tergite as observed in various representatives of Vicissicuadata (see also Lerosey-Aubril et al. 2017).

2.3. Characters removed

Char. 69 of Ortega-Hernández et al. (2013)**. Nature of differentiated preterminal tergite**
This character aimed to discriminate forms with anteriorly expanded tailspines from that with regular tailspines. The anterior expansion of the tailspine of some aglaspidids is now regarded as homologous to the pretelsonic twelfth segment of others, and is therefore regarded as a distinct state of modified Character 60.

References

Bruton, D.L., 1981. The arthropod *Sidneyia inexpectans*, middle Cambrian, Burgess Shale, British Columbia. Philosophical Transactions of the Royal Society B, Biological Sciences 295, 619–653.

Edgecombe, G.D., Paterson, J.R., García-Bellido, D.C., 2017. A new aglaspidid-like euarthropod from the lower Cambrian Emu Bay Shale of South Australia. Geological Magazine 154, 87–95.

Fortey, R.A., Rushton, A.W.A., 2009. The Ordovician aglaspidid arthropod *Tremaglaspis* reconsidered. Memoirs of the Association of Australasian Palaeontologists 37, 17–23.

Hesselbo, S.P., 1992. Aglaspidida (Arthropoda) from the Upper Cambrian of Wisconsin. Journal of Paleontology 66, 885–923.

Ivantsov, A.Yu., 1999. Trilobite-like arthropod from the Lower Cambrian of the Siberian Platform. Acta Palaeontologica Polonica 44, 455–466.

Lerosey-Aubril, R., Ortega-Hernández, J., Kier, C., Bonino, E., 2013. Occurrence of the Ordovician-type aglaspidid *Tremaglaspis* in the Cambrian Weeks Formation (Utah, USA). Geological Magazine 150, 945–951.

Lerosey-Aubril, R., Paterson, J.R., Gibb, S., Chatterton, B.D.E. 2017. Exceptionally-preserved late Cambrian fossils from the McKay Group (British Columbia, Canada) and the evolution of tagmosis in aglaspidid arthropods. Gondwana Research 42, 264–279.

Ortega-Hernández, J., Legg, D.A., Braddy, S.J., 2013. The phylogeny of aglaspidid arthropods and the internal relationships within Artiopoda. Cladistics 29, 15–45.

Ortega-Hernández, J., Van Roy, P., Lerosey-Aubril, R., 2016. A new aglaspidid euarthropod with a six-segmented trunk from the Lower Ordovician Fezouata Konservat-Lagerstätte, Morocco. Geological Magazine 153, 524–536.

Siveter, D.J., Fortey, R.A., Zhu, X., Zhou, Z., in press. A three-dimensionally preserved aglaspidid arthropod with a calcitic cuticle from the Ordovician of China. Geological Magazine.

Stein, M., Budd, G.E., Peel, J.S., Harper, D.A.T., 2013. *Arthroaspis* n. gen., a common element of the Sirius Passet Lagerstätte (Cambrian, North Greenland), sheds light on trilobite ancestry. BMC Evolutionary Biology 13, 99.

Van Roy, P., 2006. A new aglaspidid arthropod from the Upper Ordovician of Morocco with remarks on the affinities and limitations of Aglaspidida. Transactions of the Royal Society of Edinburgh: Earth Sciences 96, 327–350.

Zacaï, A., Vannier, J., Lerosey-Aubril, R., 2016. Reconstructing the diet of a 505-million-year-old arthropod: Sidneyia inexpectans from the Burgess Shale fauna. Arthropod Structure & Development 45, 200–220.
